# Response to “Comment on ‘Flavoring Chemicals in E-Cigarettes: Diacetyl, 2,3-Pentanedione, and Acetoin in a Sample of 51 Products, Including Fruit-, Candy-, and Cocktail-Flavored E-Cigarettes’”

**DOI:** 10.1289/EHP348

**Published:** 2016-06-01

**Authors:** Joseph G. Allen, Skye S. Flanigan, Mallory LeBlanc, Jose Vallarino, Piers MacNaughton, James H. Stewart, David C. Christiani

**Affiliations:** Harvard T.H. Chan School of Public Health, Boston, Massachusetts, USA

We appreciate the opportunity to respond to the letter to the editor from Pierce et al. Nowhere do Pierce et al. identify any factual errors in our work; our key findings stand. We will, however, take this opportunity to address several of the points they raised.

In their analysis comparing diacetyl in e-cigs to occupational exposure limits (OELs), Pierce et al. selectively chose to evaluate only the median (6.0 µg/e-cigarette for diacetyl and 1.6 µg/e-cigarette for 2,3-pentanedione) from our data to reinforce their point that exposures are below the OEL they derived (176 μg/day for diacetyl). In choosing only the median, Pierce et al. ignored that our study found, in a sample of only 51 of over 7,000 flavors, a flavored e-cigarette with a diacetyl concentration of 238 µg/e-cigarette, which exceeds the 176 µg/day OEL they calculated. There is additional support in the literature showing the potential to exceed their derived OEL, as well, including a paper cited by Pierce et al. Farsalinos et al. (2015) measured diacetyl directly in the liquid of e-cigarettes and then used this to estimate a daily dose (median = 6 μg/day; interquartile range: 26–278 μg/day). The 75th percentile concentration in Farsalinos et al. (2015) exceeds the daily dose limit of 176 µg/day that Pierce et al. used. Therefore, at least 25% of flavored e-cigarettes samples in the Farsalinos et al. (2015) paper would exceed the 176 µg/day limit derived by Pierce et al.

We also want to reiterate our position, stated clearly in the discussion section of our paper along with our rationale, that the use of OELs for this population is inappropriate. Our position is in agreement with NIOSH, which published a response to the paper by Farsalinos et al. (2015) in which they stated that OELS are “…not intended to establish “safe” exposure concentrations for consumers or the general public^”^ (Hubbs et al., 2015).

Pierce et al. also misrepresent the findings of their own earlier work (Gaffney et al. 2015; Pierce et al. 2014; Pierce et al. 2015) in which diacetyl and 2,3-pentanedione levels were measured. In their letter they stated that “Over the past five years, we have published the results of several studies in which diacetyl and 2,3-pentanedione levels were measured in various consumer products.” As evident by the dates in the in-line citation, all 3 of their papers cited were published within 1.5 years; they do not have a 5-year record of publishing on this topic. Further, the ‘various consumer products’ include only 2 products: cigarettes and coffee. Additionally, in their letter they directly contradict the conclusions in their earlier work. In this letter they state “Gaffney et al. (2015) and Pierce et al. (2015) found that grinding, brewing, and consuming unflavored coffee was associated with airborne diacetyl concentrations that were several times higher than the NIOSH and ACGIH short-term (0.025 and 0.020 ppm, respectively) and 8-hour (0.005 and 0.010 ppm, respectively) OELs for diacetyl.” This is inaccurate and inconsistent with their own paper published in 2015. For workers brewing coffee, they actually state the exact opposite in the Pierce et al. (2015) paper: “None of the individual short-term (15 min) barista samples (maximum of 0.01 ppm) exceeded the proposed NIOSH or ACGIH STELs (0.025 ppm and 0.02 ppm, respectively).” And for customers consuming unflavored coffee, the maximum 8-hour exposure reported in their simulation (0.005 ppm; Table 2) was below the ACGIH 8-hour limit (0.010 ppm), and it was at the NIOSH recommended exposure limit (0.005 ppm), not above it, and certainly not “several times higher” than either limit (Pierce et al. 2015).

We further note that Pierce et al. (2015) and Gaffney et al. (2014) appear not to have been peer reviewed, based on the short time between submission and publication (received, revised and accepted all in 3 and 1 days, respectively). Also, the 2 papers are on the same topic, were received by the same journal within 2 days of each other, and contain 6 identical and 12 nearly identical sentences, although only 1 discloses the funding source as being from two companies involved in diacetyl litigation. Furthermore, the exposure data reported in Pierce et al. (2015) were collected not in a coffee shop but in a small kitchen with a very low ventilation rate that we calculate to be well below the ASHRAE minimum ventilation rates for cafeterias/fast food dining of 19 ft^3^/min/person (ASHRAE 2013).

Pierce et al. attempt to minimize risks by comparing flavored e-cigarettes and coffee beans, commenting, “Unless one assumes that unflavored coffee beans pose a serious risk of ‘popcorn lung,’ a rare and oftentimes lethal disease, then one should agree that exposures to airborne diketone levels above the NIOSH and ACGIH OELs are not necessarily indicative of respiratory risk.” They do not seem to be aware that, in fact, several workers at a coffee processing workplace were recently diagnosed with bronchiolitis obliterans (“popcorn lung”), and NIOSH’s investigation found a 2.7-fold elevated standard mortality ratio for obstruction for workers at this site (Bailey et al. 2015). The NIOSH investigation concluded that, “The exposure group working in both coffee flavoring and grinding/packaging of unflavored coffee areas had significantly lower mean ratio of forced expiratory volume in 1 s to forced vital capacity and percent predicted mid-expiratory flow than workers without such exposure,” and “Current workers have occupational lung morbidity associated with high diacetyl and 2,3-pentanedione exposures, which were not limited to flavoring areas.”

Finally, Pierce et al.’s comparison of diketone exposures from e-cigarettes and cigarettes, and their assertion of an increase in diketone exposure by not switching to e-cigarettes, misses a major point. Nearly 2 million children have tried e-cigarettes, 160,000 of whom reported that they had not used cigarettes (CDC 2013). Pierce et al. stated, “Ironically, suggesting that diketone levels in e-cigarettes are potentially dangerous could actually lead to higher diketone exposures in the smoking population if smokers decide not to switch to e-cigarettes due to as yet unfounded health concerns.” What about the 160,000 children who tried e-cigarettes who had not used cigarettes? We see no irony.

In conclusion, we stand by our work and the facts presented in our paper: diacetyl and other flavoring chemicals are in many flavored e-cigarettes, including flavors, like cupcake and cotton candy, that we deem are particularly appealing to kids. Considering the history of severe and irreversible lung disease associated with some workers who inhaled diacetyl, and the similar exposure pathways for consumers of flavored e-cigarettes, it is prudent to evaluate this potential hazard further, restrict access by youth, and provide consumers with information and warnings similar to those given to workers.

## References

[r1] ASHRAE (2013). Standard 62.1-2013—Ventilation for Acceptable Indoor Air Quality..

[r2] Bailey RL Respiratory morbidity in a coffee processing workplace with sentinel obliterative bronchiolitis cases. Am J Ind Med 58 12 1235 1245 2015, doi:10.1002/ajim.22533 26523478 PMC4715657

[r3] CDC (Centers for Disease Control and Prevention) 2013 Notes from the field: electronic cigarette use among middle and high school students—United States, 2011–2012. MMWR Morb Mortal Wkly Rep 62 35 729 730, PMID: 24005229 PMC4585627

[r4] Farsalinos KE Kistler KA Gillman G Voudris V 2015 Evaluation of electronic cigarette liquids and aerosol for the presence of selected inhalation toxins. Nicotine Tob Res 17 2 168 174, doi:10.1093/ntr/ntu176 25180080 PMC4892705

[r5] Gaffney SH Abelmann A Pierce JS Glynn MH Henshaw JL McCarthy L 2015 Naturally occurring diacetyl and 2,3-pentanedione concentrations associated with roasting and grinding unflavored coffee beans in a commercial setting. Toxicol Rep 2 1171 1181, doi:10.1016/j.toxrep.2015.08.003 PMC5598149 28962459

[r6] Hubbs AF Cummings KJ McKernan LT Danvorkic DA Park RM Kreiss K 2015 Comment on Farsalinos et al., “Evaluation of electronic cigarette liquids and aerosol for the presence of selected inhalation toxins.” Nicotine Tob Res 17 10 1288 1289, doi:10.1093/ntr/ntu338 25586777

[r7] Pierce JS Abelmann A Lotter J Comerford C Keeton K Finley BL 2015 Characterization of naturally occurring airborne diacetyl concentrations associated with the preparation and consumption of unflavored coffee. Toxicol Rep 2 1200 1208, doi:10.1016/j.toxrep.2015.08.006 PMC5598504 28962462

[r8] Pierce JS Abelmann A Spicer LJ Adams RE Finley BL 2014 Diacetyl and 2, 3-pentanedione exposures associated with cigarette smoking: implications for risk assessment of food and flavoring workers. Crit Rev Toxicol 44 5 420 435, doi:10.3109/10408444.2014.882292 24635357

